# Clozapine Increases Nestin Concentration in the Adult Male Rat Hippocampus: A Preliminary Study

**DOI:** 10.3390/ijms23073436

**Published:** 2022-03-22

**Authors:** Hakan Kayir, Bryan W. Jenkins, Begüm Alural, Jibran Y. Khokhar

**Affiliations:** 1 Department of Biomedical Sciences, Ontario Veterinary College, University of Guelph, Guelph, ON N1G 2W1, Canada; hkayir@uoguelph.ca (H.K.); bjenki01@uoguelph.ca (B.W.J.); 2 Department of Molecular and Cellular Biology, University of Guelph, Guelph, ON N1G 2W1, Canada; balural@uoguelph.ca

**Keywords:** nestin, schizophrenia, animal model, clozapine, MK-801, dizocilpine, NMDA, neural stem cell, neurogenesis, antipsychotic

## Abstract

Patients with schizophrenia, and rodent models of the disease, both exhibit suppressed neurogenesis, with antipsychotics possibly enhancing neurogenesis in pre-clinical models. Nestin, a cytoskeletal protein, is implicated in neuronal differentiation and adult neurogenesis. We hypothesized that schizophrenia pathogenesis involves nestin downregulation; however, few studies have related nestin to schizophrenia. We assessed nestin protein concentration, prepulse inhibition (PPI), and social interaction in the MK-801 model of schizophrenia, with or without antipsychotic (clozapine) treatment. Adult male Sprague–Dawley rats were intraperitoneally administered saline or MK-801 (0.1 mg/kg) to produce a schizophrenia-like phenotype, with concomitant subcutaneous injections of vehicle or clozapine (5 mg/kg). PPI was assessed on days 1, 8, and 15, and social interaction was assessed on day 4. Hippocampus tissue samples were dissected for Western blotting of nestin concentration. MK-801 alone did not alter nestin concentration, while clozapine alone enhanced hippocampal nestin concentration; this effect was not apparent in animals with MK-801 and clozapine co-administration. MK-801 also produced schizophrenia-like PPI disruptions, some of which were reversed by clozapine. Social interaction deficits were not detected in this model. This is the first report of clozapine-induced enhancements of hippocampal nestin concentration that might be mediated by NMDA receptors. Future studies will explore the impact of neurodevelopmental nestin concentration on symptom onset and antipsychotic treatment.

## 1. Introduction

Nestin is a Type VI intermediate filament protein that serves as marker of neuroepithelial stem cells [1] that contributes to embryonic and adult neurogenesis. Neural stem cells (NSC) first generate as progenitor cells, and neurons and glial cells differentiate from those cells during embryonic development. Nestin gene expression is highly correlated with the first stages of neural differentiation and as NSC divide asymmetrically [2]. It is thus no surprise that nestin knockout mice have high rates of embryonic mortality; NSC cultures derived from embryos of these mice show reduced self-renewal and elevated apoptosis [3]. Interestingly, knocking out nestin in mice, or blocking its polymerization had no detectable effect on cytoskeleton integrity. Conversely, two different mouse strains engineered for nestin deficiency appear without significant reductions in neuronal development [4]. Additionally, external stress (e.g., trauma) may be necessary to activate nestin-dependent mechanisms during development [5].

Schizophrenia is a complex psychiatric disorder with a multigenic and multifactorial origin. It is generally described as a neurodevelopmental disorder, and once its symptoms appear during early adolescence or adulthood, the disease persists throughout life with debilitating effects. One of the accepted theories for the pathogenesis of schizophrenia is the “developmental risk factor” model. This model suggests that early neurodevelopmental insults are necessary but not sufficient to develop the disease state; however, the probability of psychosis leading to schizophrenia could increase when combined with environmental risk factors such as urban living, migration, and drug abuse, especially during childhood and adolescence [6]. Post-mortem immunohistochemical studies reveal patients with schizophrenia have reduced NSC counts in the dentate gyrus (DG) [7], while other studies support the involvement of neurogenesis-related abnormalities in schizophrenia pathogenesis, especially in the subgranular zone (SGZ) of the hippocampus [8]. Reduced nestin expression was previously detected in dopaminergic and glutamatergic neurons differentiated from patient-derived NSCs when compared to NSCs derived from healthy controls [9].

Rodent models of schizophrenia also reflect some of the neurogenesis-related aspects of disorder. NMDA antagonist-treated rodents also present with alterations in neurogenesis. Rats receiving phencyclidine treatment for two weeks and administered bromodeoxyuridine (BrdU) at the end of treatment show a decrease in the number of BrdU+ cells in the SGZ [10]. Similarly, a two-week treatment of MK-801 decreases the number of BrdU+ cells in the DG of C57/BL mice [11]. Furthermore, several antipsychotics can reverse the effects of these NMDA antagonists. Maeda et al. showed that a two week phencyclidine or MK-801 treatment reduces the number of BrdU+ cells, and clozapine, but not haloperidol, treatment prevents the phencyclidine-induced suppression [12]. Recently, the cytoprotective effect of clozapine and ineffectiveness of haloperidol in ketamine-induced cytotoxicity were demonstrated in NSCs obtained from the SVZ of mice [13]. Various other studies using different animal models of schizophrenia report neuroprotective effects of clozapine and other antipsychotics including risperidone, haloperidol, and olanzapine [14]. Interestingly, several studies revealed that antipsychotics may increase the number of BrdU+ cells in control animals.

These studies mostly focused on neurogenesis; however, the effects of these models and drug treatments on precursors to neurogenesis remain unknown. We hypothesize that one of the aspects of schizophrenia pathogenesis could be the downregulation of nestin expression and that clozapine, the prototypical atypical antipsychotic, could reverse this process. To investigate this, we treated rats with MK-801, with or without clozapine pre-treatments, for two weeks. MK-801 induced behavioural and biochemical changes in rats have been accepted as a valuable animal model to investigate psychosis that show strong predictive validity: antipsychotic drugs reverse the schizophrenia-like behavioural and biochemical changes [15]. Nestin concentration was quantified using Western blot analysis of the hippocampus collected 24 h after last treatments. Prepulse inhibition (PPI) of the acoustic startle reflex and social interaction tests were performed to investigate schizophrenia-like behaviour in these rats and detect any phenotypic response to antipsychotic treatment.

## 2. Results

**Figure 1:**
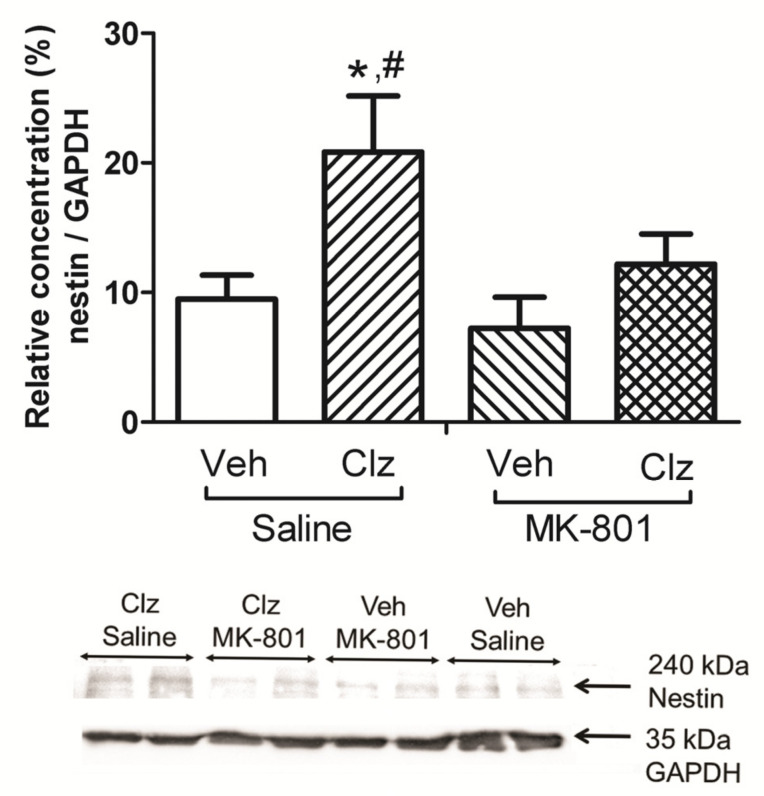
Effects of clozapine 5 mg/kg (Clz), MK-801 0.1 mg/kg, and their combinations on nestin protein concentration in hippocampus of the rats, and representative Western blot images of nestin and GAPDH proteins. Clozapine increases hippocampal nestin concentration, and this effect is blocked by MK-801 co-treatment. Nestin protein concentrations were normalized according to GAPDH loading control (* *p* < 0.05, compared to Veh + Saline group, # *p* < 0.05, compared to Veh + MK-801 group, post hoc Tukey’s test; Veh, vehicle, *n* = 6/group).

A significant effect of drug treatment on nestin protein concentration in the hippocampus was observed (F(3,20) = 4.301; *p* = 0.017; Figure 1). Post hoc tests indicated that nestin concentration increased in the clozapine-treated group compared to the control group (*p* = 0.046; Figure 1). Nestin concentration was also significantly higher than the Vehicle + MK-801 treated group (*p* = 0.014; Figure 1). 

**Figure 2:**
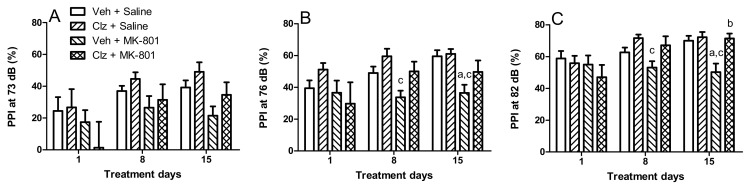
MK-801 induces pre-pulse inhibition deficits, which are ameliorated by clozapine treatment. Effects of clozapine 5 mg/kg (Clz), MK-801 0.1 mg/kg, and their combinations on prepulse inhibition (PPI) of rats on the 1st, 8th, and 15th days of the treatments at 73 (**A**), 76 (**B**), and 82 (**C**) decibel (dB) prepulse intensity levels (^a^ *p* < 0.05, compared to Veh + Saline group; ^b^ *p* < 0.05, compared to Veh + MK-801 group; ^c^ *p* < 0.05, compared to Clz + Saline group, post hoc Tukey’s tests; Veh, vehicle, *n* = 8/group).

PPI levels at all three prepulse intensities (73, 76, and 82 dB) changed significantly between the three measurements taken over the two week period (time effect: F(2,48) = 8.244, 5.445, and 9.913; *p* = 0.0001, 0.007, and 0.0001, respectively). Treatments had significant effects on PPI levels at the 76 and 82 dB prepulse intensities (treatment effect: F(3,24) = 4.228 and 3.301; *p* = 0.016 and 0.037, respectively). The effects of the treatments interacted with the measurement time only at the 86 dB prepulse intensity level (F(6,48) = 2.901; *p* = 0.017). Post hoc analyses indicated that MK-801 (0.1 mg/kg) disrupted PPI after two weeks of treatment at the 76 and 82 dB prepulse intensity levels (*p* = 0.018 and 0.006; Figure 2B,C, respectively), but not at 73 dB intensity level (Figure 2A). Clozapine (5 mg/kg) reversed the MK-801-induced PPI disruption after two weeks of treatment at the 82 dB prepulse intensity (*p* = 0.004; Figure 2C). 

**Figure 3:**
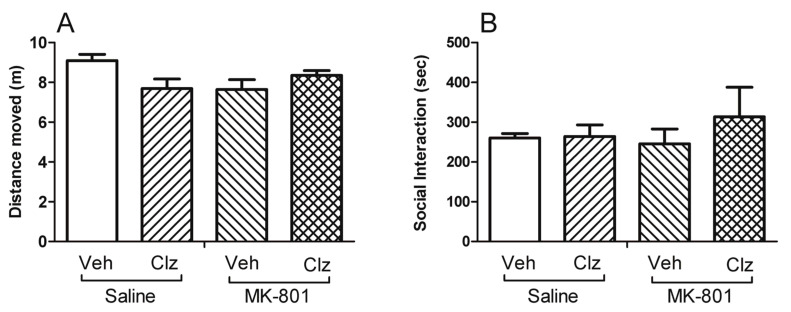
Neither MK-801 nor clozapine treatment altered motor reactivity to a novel environment or social interaction. Effects of clozapine 5 mg/kg (Clz), MK-801 0.1 mg/kg, and their combinations on motor reactivity to a novel environment (**A**), and social interaction duration (**B**) of the rats (Veh, vehicle), on the third and forth days of the treatments, respectively (*n* = 8/group).

On the third day of treatments, there was no difference in motor activity between groups (F(3,28) = 2.903; *p* = 0.052; Figure 3A), and on the following day, no difference was observed in SI duration (F(3,12) = 0.444; *p* = 0.726; Figure 3B).

## 3. Discussion

The present study shows that subchronic treatment of adult male rats with clozapine increased nestin protein concentration in the hippocampus compared to the vehicle control group. Although MK-801 did not alter nestin concentration, it prevented the clozapine-induced increase in nestin concentration when given together. To our knowledge, such an effect of clozapine on nestin concentration has not been previously reported. Moreover, MK-801 disrupted PPI, and concomitant clozapine treatment reversed this disruption. In our subchronic treatment model, the disruptive effect of MK-801 became apparent during measurements on the eighth treatment day, but the preventive effect of clozapine reached statistical significance only on the final evaluation day. Thus, the detected effects of MK-801 and/or clozapine treatments could be the result of gradual changes. In line with this perspective, we did not observe any alterations in motor activity or social interaction, ran on the third and fourth days of treatment. In the present study, the PPI test was our primary behavioural measure of interest because of its superior predictive and face validity for modeling schizophrenia compared to locomotor activity and social interaction. We did not repeat these two tests during the second week of the treatments to avoid possible confounding effects on the PPI measurements. 

There are only a few reports indicating a relationship between schizophrenia pathogenesis and nestin concentration. Robicsek et al. programmed keratinocytes obtained from patients with schizophrenia into induced pluripotent stem cells (iPSCs) and further differentiated them into dopaminergic and glutamatergic neurons. Nestin concentration was reduced in cells obtained from patients with schizophrenia. Furthermore, the intracellular distribution of nestin was different from that of the control group, as it was concentrated in the nucleus rather than the cytoplasm [9]. However, our two week treatment of MK-801 (0.1 mg/kg) did not induce any change in nestin concentration in the hippocampus, although it did produce a significant disruption in PPI. Thus, changes in nestin concentration might be not essential for MK-801 to induce its disrupting effects on PPI. The studies directly investigating nestin expression or nestin-expressing cell counts after NMDA receptor modulation are rare. Kitayama et al. showed that systemic administration of NMDA decreased, whereas MK-801 increased, nestin-, BrdU-, and PCNA-expressing cell counts only in the DG, but not in the SVZ or olfactory bulb areas in Std-ddY mice. When co-administered, NMDA reversed the MK-801 enhancements in neurogenesis [16]. Other studies exploring neurogenesis following NMDA receptor blocker administration generally report a decrease in the BrdU+ new cell formation in the DG [10,11,12]. Thus, it seems that NMDA receptors play an important role in the control of new cell proliferation in hippocampus, but it is not simple nor unidirectional. This altering effect on NMDA receptor activity is possibly true for nestin expression. 

Although we did not see a marked effect in nestin concentration after MK-801 treatment alone, this treatment reversed the effects of clozapine on hippocampal nestin. Neuroprotective- and neurogenesis-stimulating effects of clozapine are known, but the mechanism for those effects are not yet established. Our results show that clozapine, when given daily at 5 mg/kg for 15 days, appears to increase nestin protein concentration in the hippocampus. These effects of clozapine are no longer present when clozapine is co-administered with an NMDA receptor antagonist, which indicates that clozapine may have neuroprotective- and neurogenesis-stimulating effects, and these effects may be mediated directly or indirectly through NMDA receptors.

Clozapine is still accepted as the most effective treatment option for patients with schizophrenia who do not respond to other antipsychotics. It has unique clinical effects, such as decreasing suicidality in patients, and an unusual pharmacological profile, such as acting as a norepinephrine reuptake inhibitor and a norepinephrine α-2 receptor antagonist, beyond being a weak dopamine D2 receptor antagonist [17]. Clozapine effectively reverses dysfunction in animal models of schizophrenia produced by NMDA receptor antagonism, such as the MK-801-induced disruption of PPI [18], corroborated by our present findings. Furthermore, this effect of clozapine was shown in a double-blind, placebo-controlled human study wherein subanesthetic doses of ketamine exacerbated the psychotic symptoms in patients with schizophrenia while they were drug-free. When these patients were treated with clozapine, a significant suppression of ketamine-induced psychotic symptoms was observed [19]. Although these studies propose that effects of clozapine could be mediated by NMDA receptor-related mechanisms, the involvement of other receptor systems, such as serotonergic-, muscarinic-, and adrenergic-mediated indirect effects were also discussed. Several other studies propose that clozapine can directly modulate NMDA receptors. Single unit recordings of dopaminergic neurons in the ventral tegmental area of Sprague–Dawley rats indicate that intravenous administration of clozapine increased firing rates and this effect is blocked by MK-801 and reversed by L-701,324, a potent glycine site specific antagonist of NMDA receptors [20]. These results are supported by existing findings that decreasing the endogenous glutamate receptor blocker kynurenic acid, which also acts through the glycine binding site, could potentiate the effect of clozapine on dopaminergic neuron firing rates [21].

There is evidence that daily injections of clozapine for eight weeks could increase NMDA receptor binding in metabotropic glutamate receptor 5 (mGluR5) knockout mice [22]. Interestingly, chronic treatment did not alter dopamine D2, 5-HT2A, or muscarinic M1/M4 receptor binding in these mice. Thus, the reversal of behavioural abnormalities such as amelioration of the locomotor disruption and reversal of the PPI deficit by clozapine treatment was strongly associated specifically with NMDA receptors [22]. In addition to the antipsychotic effects, our results imply that increased nestin concentration could be another NMDA receptor-mediated effect of clozapine, which adds to the literature related to the interactions between clozapine and NMDA receptors.

Although nestin was first described in NSCs, it is expressed in other tissues such as bone marrow, immune system, heart, lung, kidney, gut, and vascular endothelium [23]. Among the others, vascular endothelial cells may also have been present in hippocampal tissue samples in our study. Previously, nestin expression was observed in proliferating endothelial progenitor cells (EPCs), but not in mature endothelium [24]. Moreover, the CNS-specific intronic enhancer of the nestin gene did not induce nestin expression in epithelial cells, and thus the presence of two different mechanisms that control nestin in epithelial and neuronal stem cells were proposed [24]. Angiogenesis in adult brain is under strict control that could be induced by pathological conditions such as hypoxia and carcinogenesis. The hypoxia-inducible factors and vascular endothelial growth factor (VEGF) appears to mediate most of these processes. There has been no evidence showing that clozapine can alter these factors or increase angiogenesis in the brain, thereby inducing nestin production; however, this possibility cannot be ruled out until directly tested [25].

This study was not without limitations. While we chose to use a pharmacological model of schizophrenia, we recognize that using a neurodevelopmental model may provide additional insights given the involvement of nestin in neurodevelopment and the early stages of progenitor cells differentiating into their mature cell types [2]. In offspring of pregnant C57Bl/6 mice injected with polyriboinosinicpolyribocytidylic acid (poly I:C, maternal immune activation model), which go on to develop a schizophrenia-like phenotype in adulthood, nestin is increased relative to control [26]. Another limitation is that we only used male rats for this study. Although sex differences in nestin-expressing NSCs were previously investigated in vitro and determined to be non-existent [27], evidence exists in vivo to support sex-dependent difference in rat nestin expression [28]. Considered together with existing preclinical evidence of female rats being less sensitive to the effects of clozapine [29], future studies could explore whether this differential response to clozapine administration is mediated in part through differences in nestin expression. This study was further limited in that our investigation focused solely on hippocampal nestin concentration. The hippocampus was selected as the region of interest for this because the majority of nestin-expressing NSCs are found in the hippocampus; this brain area has also been shown to be involved in neurogenesis-related abnormalities in schizophrenia patients. Taken together, the results of this study and the known localization of nestin-expressing NSCs in the SVZ and SGZ [30] necessitate future investigations of clozapine-induced changes in SVZ and SGZ nestin-expressing cell counts using immunohistochemical and RNA-based methods. Furthermore, targeting additional nestin-expressing brain regions in future studies may also help to uncover nestin’s role in neurodevelopment and neurogenesis in those regions, as well as the impact of antipsychotics on these processes. In addition, we evaluated the changes in behavioural parameters and nestin concentration in a 2 week time period. Increase in nestin concentration might start at an earlier point during clozapine treatment, and it would also be important to explore how long this increased state persists.

## 4. Materials and Methods

### 4.1. Animals and Procedure

All procedures complied with the guidelines described in the Guide to the Care and Use of Experimental Animals (Canadian Council on Animal Care, 1993) and set by the Animal Care Committee at the University of Guelph. Male Sprague–Dawley rats (Charles River, Raleigh, NC, USA), weighing 241–290 g and aged postnatal day 56 (PND56) upon arrival in the laboratory, were housed three per cage in a Tecniplast SealSafe^®^ Plus vent-rack with environmental enrichment and ad libitum access to food and water. They were maintained under a 12:12 h light/dark cycle (lights on at 7:00 a.m.) with constant ambient temperature (22 ± 2 °C) and humidity (50–70%). Behavioral tests were performed during the dark phase of the light/dark cycle. All the rats were handled daily for three days before starting the experiments. They were treated for 15 days, and 24 h after final treatments, they were decapitated, and their brains were immediately removed; hippocampi were dissected with blunt forceps on an ice-cold glass plate. The samples were stored at −80 °C until homogenizing, and nestin protein concentration was measured using Western blot analysis. Whole left and right hippocampi were used for the samples. PPI was measured on the 1st, 8th, and 15th days of treatment. On the fourth day of treatment, social interactions (SI) of rats within the same treatment group were evaluated. 

### 4.2. Drugs and Treatments

Clozapine HCl was provided by the NIMH Drug Supply Program, dissolved in 50 µL 37% HCl, and diluted with saline (0.9% NaCl) to desired volume. The pH of the solution was adjusted to approximately 6 using 1N NaOH. Vehicle was prepared using the same protocol, without the addition of clozapine. MK-801 was purchased from Abcam (ab120027) and dissolved in saline. Solutions were freshly prepared every few days. Rats were randomly assigned into four groups (*n* = 8/group) to receive either vehicle/saline, clozapine/saline, vehicle/MK-801, or clozapine/MK-801. Drugs were administered at a volume of 1 mL/kg daily between 10:00 and 11:00 a.m. Vehicle or clozapine (5 mg/kg) were administered subcutaneously (s.c.) 15 min before a subsequent intraperitoneal (i.p.) injection of either saline or MK-801 (0.1 mg/kg). Doses were selected based on observations in previous studies [31]. 

### 4.3. Prepulse Inhibition of the Acoustic Startle Reflex

PPI measurements started 10 min after MK-801 or saline administration. PPI of the acoustic startle reflex test was performed using four MedAssociates Acoustic Startle Reflex chambers (MED-ASR-PRO1). Each sound-attenuating chamber was equipped with a load cell platform and two speakers to deliver acoustic stimuli, as well as a house light and a ventilation fan. The platform calibration was performed by adjusting the gain on the load cell amplifier to 200 arbitrary units at a standard weight appropriated for rats (300 g). The limits of the load cell were −2047 to +2047 arbitrary units. Rats were restrained in a Plexiglas cylinder and mounted on top of the platform. The background noise level during the study was 70 dB sound pressure level (SPL) white noise. Prepulse stimulus levels were determined according to the background noise. One day before the test sessions, rats were habituated to the Plexiglas cylinders in the startle reflex chambers for 15 min, with background noise on and five startle alone trials applied between 5 and 10 min. The testing session began with a 5 min acclimatization period in which only 70 dB background noise was present. Then, five consecutive startle stimuli (120 dB) were administered, followed by 10 blocks of five consecutive trials, and finally five startle stimuli were applied before completing the session. Each block contains the following trials in pseudorandomized order:

i. Startle alone (120 dB);
ii. Prepulse (73 dB) and startle stimuli;
iii. Prepulse (76 dB) and startle stimuli;
iv. Prepulse (82 dB) and startle stimuli;
v. No stimulus (only background noise).

All the stimuli were applied as white noise. Startle stimuli lasted for 40 ms, and the three prepulse stimuli lasted 20 ms. Prepulse stimulus levels were selected at intensities that did not elicit a significant startle reflex when applied alone. Prepulse stimuli were applied 120 ms prior to a startle stimulus (onset to onset). The inter-trial interval varied randomly between 15 and 30 s. PPI was defined as a decrease in the amplitude of the startle reflex following a prepulse stimulus and was calculated for each of the three different prepulse intensities by using the following formula:

(1) $$\%\mathrm{PPI}=100\;-\;\left(\frac{\mathrm{average}\;\mathrm{startle}\;\mathrm{reflex}\;\mathrm{in}\;\mathrm{presence}\;\mathrm{of}\;\mathrm{prepulse}\;\mathrm{stimuli}\;\text{×}\;100}{\mathrm{average}\;\mathrm{startle}\;\mathrm{reflex}\;\mathrm{without}\;a\;\mathrm{prepulse}}\right)\;$$

Each value used in the calculations were the average of 10 arbitrary units recorded at each trial. 

### 4.4. Social Interaction Test

The SI test started 15 min after MK-801 or saline administration. The SI test arena was a Plexiglas cage (100 × 100 × 40 cm) with a gray floor and black walls. It was recorded via a GigE camera mounted 210 cm from the floor, and a PC running video tracking software (Ethovision v15.0.1418, Noldus, NL, USA). The rats were familiarized with the test arena for 15 min one day before the actual test. The familiarization session started 15 min after the last injection, and the distance moved (m) was calculated for each rat to show motor reactivity to a novel environment. The arena was cleaned thoroughly using H_2_O_2_ solution before each test. On the SI test day, each rat was paired with a rat from the same treatment group. The maximum difference between body weights of the rat pairs was ±15 g. The pairs were from different cages and were not previously housed together. The neck and upper dorsal areas of rats in each pair were dyed using a non-toxic paint, one with green and the other red, which allowed the video tracking system to continuously track and distinguish the rats. The rat pairs were simultaneously placed into opposite corners of the SI test arena before recording started. When rats were less than 20 cm from each other, the tracking software began measuring SI and when they receded more than 25 cm apart, SI tracking ended. SI duration, average distance between the rats (cm), and total distance moved (m) during the SI were analyzed for 15 min.

### 4.5. Western Blotting

Quantification of nestin protein in the hippocampus was performed by Western blotting. Frozen brain areas were thawed and lysed using Triton X-100 lysis buffer (Thermo Fisher, NJ, USA), protease inhibitors (cOmplete, Mini Protease Inhibitor Cocktail, Roche, Germany), and a blade tissue homogenizer. After centrifugation, reducing buffer with β-mercaptoethanol was added to the supernatant. Protein samples were heated to 95 °C for 5 min for denaturation. GAPDH was blotted as the loading control. The polyacrylamide gels (8%) were prepared using Bio-Rad (Hercules, CA, USA) standard gel recipes. Protein samples (20 µg) were loaded onto the gel in a Bio-Rad Mini-PROTEAN Tetra Cell apparatus for 80 min at 150 V in running buffer and then transferred to a polyvinylidene difluoride (PVDF) membrane using the Bio-Rad Mini Trans-Blot^®^ Cell transfer system (80 min at 350 mAmp). The membranes were subsequently blocked with 5% bovine serum albumin (BSA, Sigma, St. Louis, MO, USA) in TBST for 60 min. Membranes were then incubated in nestin primary antibody (Bio-Techne Canada, Toronto, ON, Canada, #MAB2736) at 1:1000 dilution overnight, and incubated in the secondary antibody (Rio-Rad Goat Anti-Mouse IgG-HRP Conjugate, #1706516) at a dilution of 1:25,000 for 1 h. Imaging was performed in a ChemiDoc XRS+ Imaging System (Bio-Rad) after incubating the membranes for 2 min in UltraScence Western Substrate (FroggaBio, Concord, ON, Canada) for chemiluminescence. The bands were quantified using ImageJ software (NIH, Bethesda, MD, USA) and normalized to GAPDH concentration. All the samples were studied duplicate or triplicate. The average value of the replicated samples was used for the statistical analyses. 

### 4.6. Statistical Analysis

A Kolmogrov–Smirnov test was performed, and the histograms were visually inspected to analyze the normal distribution of continuous variables. The effects of the subchronic clozapine (5 mg/kg) and/or MK-801 (0.1 mg/kg) treatments on PPI, startle reactivity, and gross motor activity were evaluated using a repeated measures two-way analysis of variance (ANOVA) test (treatment groups × measurement time), followed by Bonferroni test for post hoc comparisons. The analyses for PPI were performed separately at three different prepulse levels (73, 76, and 82 dB). The effects of the treatments on motor reactivity to novel environment, social interaction time, and nestin protein concentration were compared with a one-way ANOVA test followed by Tukey’s test for post hoc comparisons. The level for statistical significance was set at *p* < 0.05.

## 5. Conclusions

To conclude, we have produced the first-ever report of clozapine-induced enhancements of hippocampal nestin concentrations and have suggested that this may be mediated by NMDA receptors. This work may reframe our consideration of nestin concentration in the pathogenesis of schizophrenia from a marker to a driving factor in aberrant neuronal differentiation, as well as inspire future investigations into the involvement of nestin-related neurogenetic mechanisms in atypical antipsychotic action.

## Data Availability

Data are available on request from the authors.
